# Theoretical Prediction of Thermal Expansion Anisotropy for Y_2_Si_2_O_7_ Environmental Barrier Coatings Using a Deep Neural Network Potential and Comparison to Experiment

**DOI:** 10.3390/ma17020286

**Published:** 2024-01-05

**Authors:** Cameron J. Bodenschatz, Wissam A. Saidi, Jamesa L. Stokes, Rebekah I. Webster, Gustavo Costa

**Affiliations:** 1 Environmental Effects and Coatings Branch, NASA John H. Glenn Research Center at Lewis Field, Cleveland, OH 44135, USA; jamesa.l.stokes@nasa.gov (J.L.S.); rebekah.webster@nasa.gov (R.I.W.); gustavo.costa@nasa.gov (G.C.); 2 National Energy Technology Laboratory, Pittsburgh, PA 15236, USA; wissam.saidi@netl.doe.gov; 3 Mechanical Engineering and Materials Science, University of Pittsburgh, Pittsburgh, PA 15261, USA

**Keywords:** environmental barrier coatings, thermodynamics, density functional theory, molecular dynamics, machine learning

## Abstract

Environmental barrier coatings (EBCs) are an enabling technology for silicon carbide (SiC)-based ceramic matrix composites (CMCs) in extreme environments such as gas turbine engines. However, the development of new coating systems is hindered by the large design space and difficulty in predicting the properties for these materials. Density Functional Theory (DFT) has successfully been used to model and predict some thermodynamic and thermo-mechanical properties of high-temperature ceramics for EBCs, although these calculations are challenging due to their high computational costs. In this work, we use machine learning to train a deep neural network potential (DNP) for Y_2_Si_2_O_7_, which is then applied to calculate the thermodynamic and thermo-mechanical properties at near-DFT accuracy much faster and using less computational resources than DFT. We use this DNP to predict the phonon-based thermodynamic properties of Y_2_Si_2_O_7_ with good agreement to DFT and experiments. We also utilize the DNP to calculate the anisotropic, lattice direction-dependent coefficients of thermal expansion (CTEs) for Y_2_Si_2_O_7_. Molecular dynamics trajectories using the DNP correctly demonstrate the accurate prediction of the anisotropy of the CTE in good agreement with the diffraction experiments. In the future, this DNP could be applied to accelerate additional property calculations for Y_2_Si_2_O_7_ compared to DFT or experiments.

## 1. Introduction

Silicon carbide (SiC)-based ceramic matrix composites (CMCs) hold strong promise for gas turbines in both aerospace and power generation applications, as they have higher temperature capability and lower density than currently prevalent nickel-based superalloys. However, SiC-based CMCs are prone to degradation from oxygen and water vapor at high temperatures via the formation of a silica thermally grown oxide scale and subsequently volatile Si(OH)_4_, which leads to the recession of the component [1,2]. As such, environmental barrier coatings (EBCs) are necessary for CMC engine components, as they can match the high temperature capabilities and mitigate water vapor-induced corrosion.

Silicate materials, including materials such as mullite and the rare-earth disilicates (RE_2_Si_2_O_7_), are currently the most highly regarded EBCs due to their good chemical resistance to oxidation and thermal expansion match with the underlying substrate. Yttrium disilicate (Y_2_Si_2_O_7_) is especially highly regarded as it has a similar bulk coefficient of thermal expansion (CTE) as the SiC-based CMCs (~4.5–5 × 10^−6^ K^−1^), which prevents cracking and subsequent spallation due to thermal cycling under engine operating conditions. However, Y_2_Si_2_O_7_ EBCs are subject to several failure mechanisms such as water vapor-induced volatility, mechanical erosion/foreign object damage, and thermochemical degradation due to reactions with calcia–magnesia–alumina–silicates (CMAS) ingested by the engine. Phase transitions in the Y_2_Si_2_O_7_ crystal structure also occur over the range of engine operating temperatures, increasing the likelihood of mechanical failures due to volumetric changes [3].

To effectively design new EBC materials, the thermodynamic and thermomechanical properties of candidate EBCs must be well understood. To this end, approaches including empirical modeling and theoretical modeling such as Density Functional Theory (DFT) have been shown to have predictive capabilities for candidate EBC materials and properties of interest [4,5,6,7,8,9]. While informative, both modeling approaches have limitations. Empirical models generally describe trends well but can have limited quantitative accuracy or fail to describe edge cases appropriately [10,11,12]. DFT, on the other hand, can have a large computational cost due to the vast chemical space of possible EBCs and the range of length scales needed to calculate properties of interest. This is especially true with the recent focus on so-called “high-entropy” ceramics, which can utilize five or more unique cations within the silicate structure [13,14].

CTE is an essential design parameter for EBC development to effectively reduce thermomechanical stresses upon heating and cooling in an engine cycle. Several studies have demonstrated that the mean CTE in Si–O bonds in silicate minerals is ~0.0 K^−1^, indicating that the primary expansion occurs in the RE–O bonds in RE silicates [11,15,16,17,18,19,20,21,22]. Hazen and Prewitt developed an empirical model for the expansion of metal–oxygen bonds valid for oxides and silicates [10]. However, this model utilizes the Pauling bond strength, *z*/*p*, where *z* is the cation charge and *p* is the cation coordination number, as the predictor of thermal expansion, *α*, and does not account for the cation ionic radius. Yttrium disilicate can have several phase transformations as a function of temperature: α-Y_2_Si_2_O_7_ (space group P$\bar{1}$), β-Y_2_Si_2_O_7_ (space group C2/m), γ-Y_2_Si_2_O_7_ (P2_1_/c), and δ-Y_2_Si_2_O_7_ (Pna2_1_). The α-phase contains four unique cation crystallographic sites: three with a coordination number of 8 and one with a coordination number of 6. The β- and γ-phases each contain one unique cation crystallographic site with a coordination of 6, while the δ-phase contains two unique cation sites each with a coordination of 7. Therefore, the Hazen and Prewitt model is unable to predict variations in the CTE between the β- and γ-phases. Similarly, the Hazen and Prewitt model would be unable to predict variations in the CTE between systems with different cations with the same coordination number.

Alternatively, a support vector regressor model using radial basis functions (SVR_RBF_) was developed by Ayyasamy and coworkers [4]. This model was trained on DFT data to predict the CTE of rare-earth disilicates (RE_2_Si_2_O_7_). It was successful at predicting CTEs for materials that are not available from experimental data, including Sm_2_Si_2_O_7_. Their model was not trained to predict the anisotropy of the CTE, only the apparent bulk CTE (ABCTE). Additionally, models such as this are generally limited to the prediction of one quantity for one class of materials.

Thermal properties can be calculated using the harmonic approximation to calculate the phonon vibrational modes. These phonon vibrational modes can be used to calculate thermal properties extrapolated to finite temperatures using the quasi-harmonic approximation. Such calculations can be crucial for determining the properties of RE disilicates, as thermal properties have been shown to be primarily driven by crystal structure bonding rather than RE cation species [11,23,24]. These calculations are computationally expensive and generally give the CTE on a volume basis rather than per lattice direction. However, it is important to determine the CTE anisotropy (i.e., on a per-lattice direction basis) for coating design, so alternative methods are required. These values can be calculated from molecular dynamics simulations at the desired temperature.

Classical atomistic simulations and even machine learning approaches are possible alternatives to expand the capabilities of theoretical simulations for EBC design [4,25]. Atomistic molecular dynamics simulations require fewer computational resources than DFT on a per-atom basis and are capable of being used to compute a variety of interesting and useful chemical properties. However, to our knowledge, there are no suitable atomistic potentials for rare-earth disilicates. As such, in this work, we use a machine learning deep neural network approach to build an atomistic potential for classical molecular dynamics simulations of Y_2_Si_2_O_7_.

To reduce the computational cost of the DFT calculations, significant recent efforts to develop machine learning models to accelerate structure and property calculations have been reported. Several excellent review articles of machine learning approaches for materials have been published in recent years [25,26,27,28,29,30,31,32]. Multiple machine learning approaches have been implemented to calculate the material properties from a variety of descriptor feature vectors as inputs. Supervised learning regression models are particularly popular to calculate a single property for a wide chemical space of materials, such as SchnetPack [33], MEGNET [34], and CGCNN [35]. Another approach is to use machine learning to fit a (deep) neural network potential (DNP) for atomistic simulations, which enables the calculation of a variety of properties usually for a single material depending on the type of MD simulation. Several software packages to develop DNPs exist, including amp [36], ænet [37], ANI [38], M3GNet [39], ALIGNN [40], and DeePMD [41], among others. These packages generally deviate in their descriptor representation of the chemical system. For crystal lattices, popular representations include atom-centered symmetry functions (ACSFs) [42,43], smooth overlap of atomic positions (SOAPs) [44], and graph-based feature representations [35]. An extensive review of chemical representations for machine learning was recently published [45].

These machine learning advancements show promise to greatly accelerate the calculation of material properties and the discovery of new materials for a variety of applications. DNPs have been demonstrated to obtain near-DFT accuracy in a range of material systems [46,47,48,49,50,51]. They have also been shown to compare well in property calculations with other methods of approximating the potential energy surface in system sizes that are cost-prohibitive in DFT, such as empirical interatomic potentials [12,52] and cluster expansions [53,54]. In this work, we implement one of these methods, the machine learning-based fitting of a DNP for a promising EBC candidate, yttrium disilicate. We show that the DNP can correctly predict the structures and properties for the three primary crystal phases of Y_2_Si_2_O_7_: β-Y_2_Si_2_O_7_, γ-Y_2_Si_2_O_7_, and δ-Y_2_Si_2_O_7_. We discuss the use of the DNP to perform the calculations of the properties that are intractable with DFT and for which no known classical MD potentials exist. Finally, to test the transferability of the potential, we calculate the crystal structure and thermal properties for a structure that was not included in the DNP training dataset: α-Y_2_Si_2_O_7_.

## 2. Materials and Methods

### 2.1. Computational Methods

#### 2.1.1. Density Functional Theory Simulations

Baseline first-principles simulations were performed using the Vienna Ab-Initio Simulation Package (VASP, version 5.4.4) implementation of plane-wave density functional theory [55,56,57,58]. These calculations were employed with a plane-wave energy cutoff of 520 eV and a *k*-space integration mesh of 1500 k-points per reciprocal atom. Projector-augmented wave (PAW) pseudopotentials were used to model the core electrons [59,60,61]. Valence electrons were modeled in some calculations with the Perdew–Burke–Ernzerhof (PBE) exchange–correlation functional [62,63], while others used the PBE modified for solids (PBEsol) functional. Initial geometries were obtained from the Materials Project database and were optimized to a maximum residual force tolerance of 1 × 10^−4^ eV/Å on any individual atom.

#### 2.1.2. Deep Neural Network Interatomic Potential Training

The DeePMD-kit package (version 2.1.0) was utilized to generate the machine learning-based atomistic potential [41]. DFT ab-initio molecular dynamics simulations were similarly performed in VASP to generate the training data for the DeePMD-kit. However, the plane-wave cutoff energy was decreased to 400 eV and *k*-space mesh with a 0.24 Å^−1^ spacing between *k*-points that was found to be sufficient [50]. The PBE exchange–correlation functional was used in these calculations. The database was constructed iteratively using adaptive training as performed in previous studies [47,49,50,51,64]. Briefly, using the first iteration of the dataset, we generated three randomly seeded DNPs, sampled 100 ps MD trajectories based on isothermal–isobaric (NPT) ensembles at various temperatures from 300 K to 2000 K, and selected configurations for further training that deviated from a set force criterion (~0.2 eV/Å). We carried out DFT calculations on the selected structures and used the additional data to enhance the dataset, and then generated the second iteration of DNPs. The adaptive training was continued until no new configurations were identified in this approach. In total, we have around 14,000 configurations for the primitive unit cell of the β-phase, 4000 configurations for the primitive unit cell of the γ-phase, and 1500 configurations for the primitive unit cell of the δ-phase. While more configurations of β-Y_2_Si_2_O_7_ were included than γ- or δ-Y_2_Si_2_O_7_, we do not expect the model to be biased to a particular crystal structure. Rather, we expect that the inclusion of more configurations of the β-phase to lead to lower variance in the predicted forces for that structure relative to the others. We note that no configurations for the α-phase were included in the training data. Additionally, the database included 3000 configurations obtained with the 2 × 2 × 2 supercell. 

The deep neural network potentials (DNPs) were developed with the DeepPot-SE method [65] as implemented in DeePMD-Kit [41]. A cut-off radius of 7 Å was used for neighbor searching, and 2 Å was set for the distance in which the smoothening starts. The embedding net was set to be 25 × 50 × 100 and the fitting net to be 240 × 240 × 240. The neural network was trained using the Adam stochastic gradient descent method that decreases exponentially from 0.001. The loss function prefactors for the energy, forces, and virials were kept at constant values of 1, 10,000, and 10, respectively. These parameters were found to be adequate and appropriate in our previous studies. The atomistic calculations using the DNP were carried out using LAMMPS.

#### 2.1.3. Molecular Dynamics Simulations

Classical molecular dynamics (MD) simulations were performed using the Large-scale Atomic/Molecular Massively Parallel Simulator (LAMMPS, version stable_29Sep2021_update3) [66]. MD simulations were run using supercells of 1 × 1 × 1 (β, γ: 22 atoms; δ: 44 atoms), 2 × 2 × 2 (β, γ: 176 atoms; δ: 352 atoms), 3 × 3 × 3 (β, γ: 594 atoms; δ: 1188 atoms), and 5 × 5 × 5 (β, γ: 2750 atoms; α, δ: 5500 atoms) multiples of the conventional unit cells for each crystal phase. Simulations were performed in four steps, using a 0.002 ps (2 fs) timestep for all steps. Firstly, a structure minimization was performed allowing all cell lattice parameter and angle degrees of freedom to vary using the conjugate gradient algorithm until the energy was converged to a tolerance of 1 × 10^−4^ eV. Secondly, an equilibration run in the canonical (NVT) ensemble was performed for 100,000 timesteps (200 ps). Thirdly, an equilibration run in the microcanonical (NVE) ensemble was performed for 100,000 timesteps (200 ps). Fourthly, a production run in the isothermal–isobaric (NPT) ensemble was performed for 1,000,000 timesteps (2 µs). Temperature and pressure were maintained with the Nosé–Hoover thermostat and barostat, respectively, with a temperature damping constant of 0.01 ps in the equilibrium runs and 10 ps in the production run and a pressure damping constant of 1000 ps. Finally, a production run in the NVE ensemble was performed for 1,000,000 timesteps (2 µs) to confirm full equilibration of the system. There was negligible difference in the calculated bond lengths and radial distribution functions between the NPT production trajectory and the NVE production trajectory, providing further evidence that the MD simulations were fully equilibrated.

#### 2.1.4. Phonon Calculations

Phonon and thermodynamic property calculations were performed using the finite-displacement approach as implemented in the Phonopy package (version 2.31.1) [67]. A 2 × 2 × 2 supercell was used for all phonon calculations. The quasi-harmonic approximation as implemented in Phonopy was used to model thermodynamic properties at non-equilibrium unit cell volumes [68]. The PhonoLAMMPS (version 0.8.3) package [69] was used to produce the Phonopy results using the DNP and LAMMPS as the force constant calculator.

### 2.2. Experimental Methods

**Figure 1:**
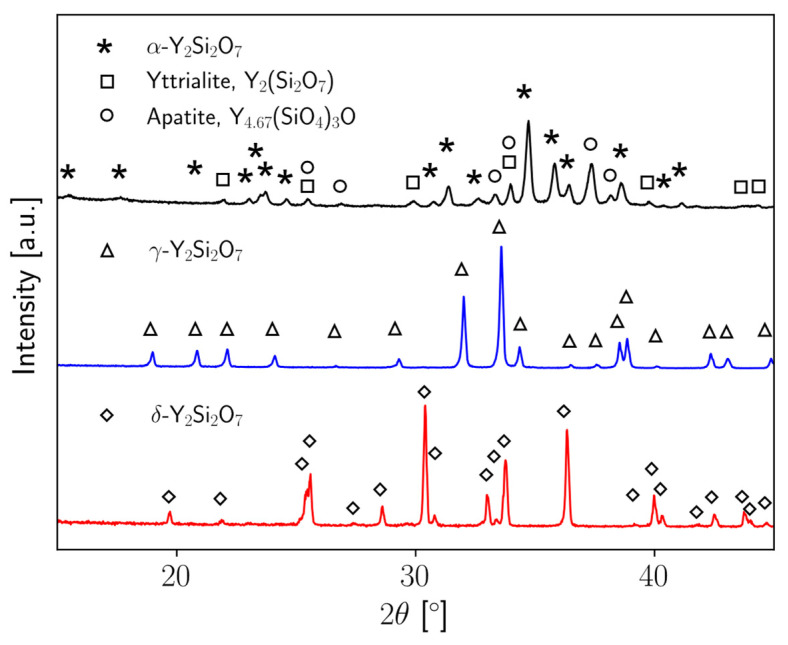
Powder X-ray diffraction scans for α- (black), γ- (blue), and δ-Y_2_Si_2_O_7_ (red) powders obtained at room temperature. γ- and δ-phase powders were single phase and corresponding peaks are indicated by triangle and diamond symbols, respectively. For the α-Y_2_Si_2_O_7_ scan, the triclinic α-phase is indicated by star symbols, the yttrialite phase is indicated by square symbols, and the apatite phase is indicated by circle symbols. β-phase CTE data were obtained from the high-temperature scans of the δ-phase sample [24].

γ-Y_2_Si_2_O_7_ and δ-Y_2_Si_2_O_7_ were previously synthesized in an earlier manuscript by the authors [24]. Briefly, Y_2_Si_2_O_7_ spray-dried powder obtained from Praxair, Inc. (99%) was heat-treated at 1600 °C for 10 h resulting in single-phase γ-Y_2_Si_2_O_7_. To obtain single-phase δ-Y_2_Si_2_O_7_, the as-received powder was heat-treated at 1700 °C for 5 h. α-Y_2_Si_2_O_7_ was synthesized for this study using a sol–gel method. All precursors were obtained from Alfa Aesar (>99%). Yttrium nitrate hydrate (Y(NO_3_)_3_·6H_2_O) and tetraethyl orthosilicate (Si(OC_2_H_5_)_4_, TEOS) were used as precursors. Y(NO_3_)_3_·6H_2_O was mixed with deionized water, and TEOS was mixed with ethanol in separate containers. The two containers were then stirred together with hydrochloric acid (HCl) to produce a gel, which was dried overnight at 60 °C and to produce a powder. The powder was then calcined at 1100 °C for 8 h to obtain α-Y_2_Si_2_O_7_. X-ray diffraction (XRD) scans of all three synthesized powders are displayed in Figure 1.

Differential scanning calorimetry (DSC) was utilized to measure the specific heat capacity (*C_P_*) of the γ-, δ-, and α-Y_2_Si_2_O_7_ synthesized powders [70]. The experiments were performed on a Netzsch STA 409 instrument using platinum crucibles with lids. An empty crucible was heated with the same temperature program to obtain a temperature calibration curve, and a sapphire standard was run to obtain a baseline curve for measurement. Sample powders were weighed out between ~20 to 50 mg so that the mass of the sample was similar to the mass of the standard. The powders were placed into the platinum crucibles and pressed into compacts before analysis. The powders were measured from room temperature to 1000 °C at a heating rate of 10 °C/min under flowing nitrogen at 50 mL/min.

CTEs for β-Y_2_Si_2_O_7_, γ-Y_2_Si_2_O_7_, and δ-Y_2_Si_2_O_7_ were measured and reported in our previous publication [24], with the experiments described in detail elsewhere [23,24]. CTEs for α-Y_2_Si_2_O_7_ were measured for this manuscript. In brief, an Empyrean diffractometer (Malvern Panalytical, Worcestershire, UK) was utilized to carry out in situ XRD scans of each powder from room temperature to 1500 °C with an Anton Paar HTK2000N heating stage. A standard CeO_2_ powder obtained from the National Institute of Standards Technology (NIST) was used to conduct the temperature calibration on the instrument. The lattice parameters at each temperature were estimated using Rietveld refinement in TOPAS software (Bruker Corporation, Billerica, MA USA, version 5) [71]. CTEs for each lattice direction were calculated as the slope of ΔL/L_0_ vs. ΔT. The average bulk CTEs of the materials were estimated as 1/3 of the unit cell volumetric expansion (ΔV/V_0_ vs. ΔT).

## 3. Results and Discussion

### 3.1. Lattice Parameters—Equilibrium Volume

A zero-pressure, 0 K geometry optimization was performed using the atomistic potential to compare to the DFT-optimized crystal cell. These results are shown in Table 1. The lattice constants calculated with both the PBE and PBEsol functionals are in good agreement with the lattice parameters determined experimentally from the X-ray diffraction data [72,73,74,75,76,77,78,79]. The experimental values were all taken at atmospheric pressures, which should have a negligible effect on the agreement between the simulated and experimental results. However, there were slight differences between the two functionals. The PBEsol-predicted lattice constants were slightly shorter than the experimental data in most cases, while the PBE-predicted lattice constants were slightly longer than the experimental data.

### 3.2. Bond Distances and Radial Distribution Functions

**Figure 2:**
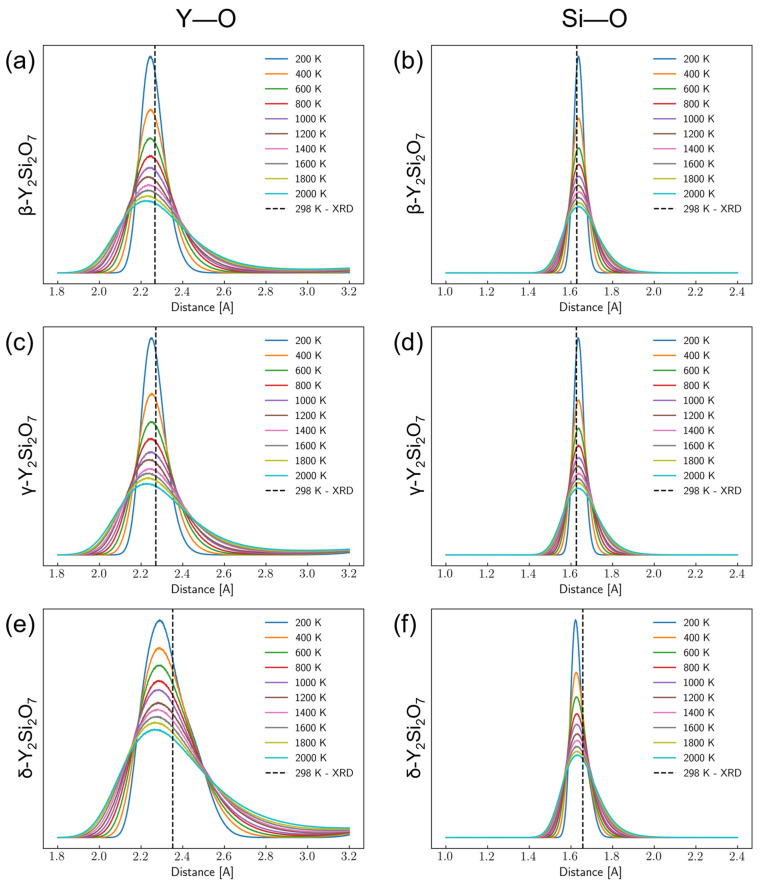
Radial distribution functions for Y–O and Si–O bonds from 200 K to 2000 K for the Y–O bonds in the (**a**) β-, (**c**) γ-, and (**e**) δ-phases of Y_2_Si_2_O_7_ as well as the Si–O bonds in the (**b**) β-, (**d**) γ-, and (**f**) δ-phases of Y_2_Si_2_O_7_ as extracted from molecular dynamics simulations using the developed interatomic potential. The vertical dashed lines are the bond lengths determined from XRD experiments at room temperature.

Since Y and Si only coordinate directly with O, the Y–O and Si–O radial distribution functions (RDFs) were calculated for each of the 5 × 5 × 5 supercell MD trajectories every 200 K from 200 K to 2000 K. These results are shown in Figure 2. As expected, tall, narrow peaks are indicated in the RDF plots at ~2.2 Å for Y–O and ~1.6 Å for Si–O, matching up with their equilibrium bond lengths. Subsequent peaks at longer distances indicate the crystalline nature of these materials (see Figures S1–S3 in the Supplementary Materials). Breadth in these peaks is due to thermal fluctuations. It can also be seen from Figure 2 that as the temperature increases, the peak height decreases and the width increases. This is likely due to a “smearing” of interatomic distances due to thermal fluctuations averaged over the trajectory. While there is some loss in peak definition at further distances with the increasing temperature, there is not an indication of melting since peaks still exist. This agrees with the melting temperature of Y_2_Si_2_O_7_, which is ~1830 °C (~2100 K) [80].

The Y–O and Si–O peaks for β-Y_2_Si_2_O_7_ and γ-Y_2_Si_2_O_7_ match very well with the results obtained from diffraction experiments at 298 K. However, the peaks for δ-Y_2_Si_2_O_7_ (Figure 2e,f) are slightly lower than the experiment. This is potentially due to the large spread in bond distances for these bonds in the δ-phase and the homogenization of these interactions during the training of the pairwise DNP. Since the DNP is a pairwise potential, it is likely that these bonds are homogenized to some degree during training, leading to a smaller spread in the distribution of bond lengths. This is also possibly indicative of the lack of influences considered in classical MD such as, i.e., electronic structure effects. While there is some quantitative disagreement between the experimental and simulation results, the qualitative trends in bond expansion are generally captured well. We are confident that the analyzed bonds remained consistent throughout the simulation as no hydrogen atoms were visually observed rotating into a new symmetrically equivalent site over the course of a trajectory, as all remained in their initial sites.

### 3.3. Thermal Properties

**Figure 3:**
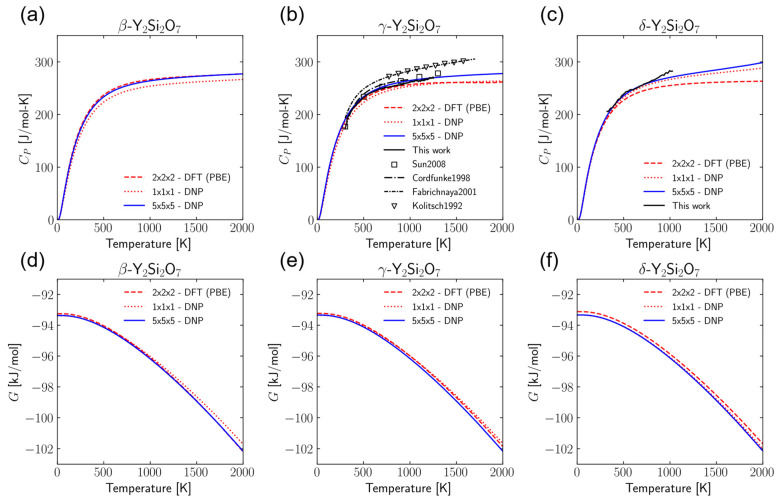
Constant pressure heat capacities (*C_P_*) and Gibbs free energy (*G*) for β- ((**a**) and (**d**), respectively), γ- ((**b**) and (**e**), respectively), and δ-Y_2_Si_2_O_7_ ((**c**) and (**f**), respectively) calculated from DNP finite-displacement phonon calculations compared to DFT and experimental results, including Sun2008 [86], Cordfunke1998 [87], Fabrichnaya2001 [88], and Kolitsch1992 [89].

Gibbs free energies (*G*) and constant pressure heat capacities (*C_P_*) were calculated using the quasi-harmonic approximation for all three phases of Y_2_Si_2_O_7_. These results are shown in Figure 3. These values were calculated for four supercell sizes, *N* × *N* × *N,* where *N* = 1, 2, 3, or 5 and compared to the DFT results utilizing either the PBE or PBEsol functional. These values of *N* were selected for direct comparison to the DFT results and due to the previous literature examples using similar supercell sizes for DFT phonon calculations [5,8,81,82,83,84,85]. While the calculation of additional properties may require even larger supercells, the errors in the properties calculated in this work were seen to have reasonably converged within this range of supercell sizes. The full set of results is shown in the Supplementary Materials in Figures S4–S9. There is very good agreement between all supercell sizes and the DFT-PBE results except for the 1 × 1 × 1, where it is likely that finite-size effects are caused by interactions between atoms and their own periodic images. There is deviation between the DNP results and DFT-PBEsol results; however, the agreement between the DNP results and DFT-PBE is quite good. Since the DNP was trained on DFT-PBE training data, this deviation is more indicative of the difference between DFT-PBE and DFT-PBEsol than any shortcomings of the DNP. For the γ-phase, the *C_P_* is larger for the cases where *N* = 2, 3, and 5 than the DFT-PBE results (Figure 3b). However, the DNP results in those cases are actually closer to the experimental values obtained from the literature [86,87,88,89]. This provides some evidence that using the larger unit cells in the DNP calculations can improve the *C_P_* calculation results. Additionally, we see in Figure 3c that for the δ-phase, the DNP does a better job of predicting the experimental results than DFT. The reduction in computational expense using the DNP compared to DFT allows for increased supercell sizes to minimize the finite-size effects for phonon frequency calculations, and it can also enable the calculation of additional properties such as diffusion constants at low gas concentrations (assuming the DNP is appropriately trained).

### 3.4. Anisotropic Coefficients of Thermal Expansion

**Figure 4:**
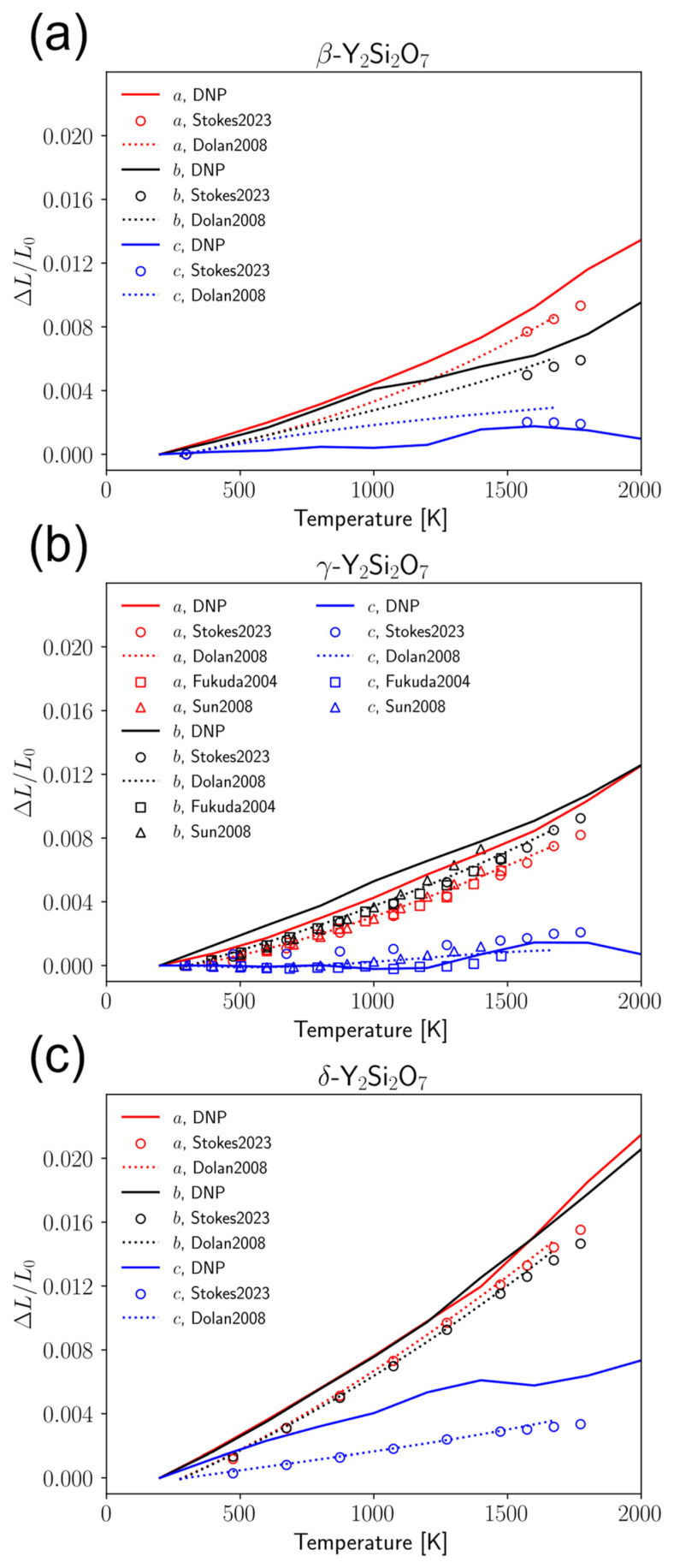
Thermal expansion of (**a**) β-, (**b**) γ-, and (**c**) δ- Y_2_Si_2_O_7_ calculated from DNP trajectories. The dotted curve represents the fitted model from Dolan et al. [73]. Square, circle, and triangle markers represent experimental data from Stokes et al. [24], Fukuda et al. [76], and Sun et al. [86], respectively.

As stated in the Introduction, the CTE is an important design criterion for EBC design. Because these materials can exhibit anisotropy in thermal expansion, it is necessary to obtain the lattice direction-dependent CTE components rather than a bulk, volumetric CTE. To this end, we used the potential to calculate the coefficient of thermal expansion (CTE) of the three predominant crystal phases of Y_2_Si_2_O_7_ to compare with those in the literature. Y_2_Si_2_O_7_ has specifically been shown in the literature to have anisotropic thermal expansion [24,73,76]. Therefore, it is necessary to adequately describe the CTE for each of the lattice directions. Several literature sources provide the mean linear CTE or bulk CTE, which assumes isotropic expansion over the temperature range. Plots showing the lattice expansion in the *a*, *b*, and *c* lattice directions along with comparison to the experimental and literature data are shown in Figure 4 and Table 2. Additionally, comparisons of the CTE values calculated at the different supercell sizes are available in the Supplementary Materials in Figures S10–S12. While there are slight quantitative deviations between the calculated data and experimental data, the general qualitative agreement is good between the DNP and experiment. It is likely that this deviation could further be reduced by increasing the size of the supercell or the simulated time.

The slopes of the curves in Figure 4 give the CTE for each of the lattice directions. Here, we calculated the CTE in each lattice direction for each crystal phase of Y_2_Si_2_O_7_ and found that the calculated values matched well with the experimental results. A comparison of the calculated and experimental CTEs is shown in [24,73,76,86,90]. In the β- and *γ*-phases (Figure 4a,b), respectively), the CTE in the *a*- and *b*-directions was overpredicted and the CTE in the *c*-direction was underpredicted. However, in the *δ*-phase (Figure 4c), the potential overpredicts the CTE in all three lattice directions. We note here that even though the DNP struggled to some degree to predict the interatomic bond lengths, as seen in Figure 2, these errors generally average out over the span of the larger crystal supercell from the phonon and CTE calculations, leading to generally good agreement in these properties of interest.

### 3.5. Transferability

To inspect the transferability of the DNP, we calculated the crystal lattice parameters, heat capacity, Gibbs free energy, and CTE of a crystal structure that was not included in the DNP training dataset: α-Y_2_Si_2_O_7_. These results are shown in Table 3. While inclusion of the α-Y_2_Si_2_O_7_ structure in the training dataset would likely lead to a more accurate model, it is not always feasible to include every possible crystal structure in the training data, whether due to computational resource limitations, structures being previously unknown, etc. Therefore, we wanted to test the DNP on an unseen structure. Interestingly, the DNP-predicted 0 K crystal structure lattice parameters for α-Y_2_Si_2_O_7_ were closer to the experimental results from Dolan et al. [73] than the experiments performed as part of this work. However, the discrepancies are small between the lattice parameters from all three sets of data.

**Figure 5:**
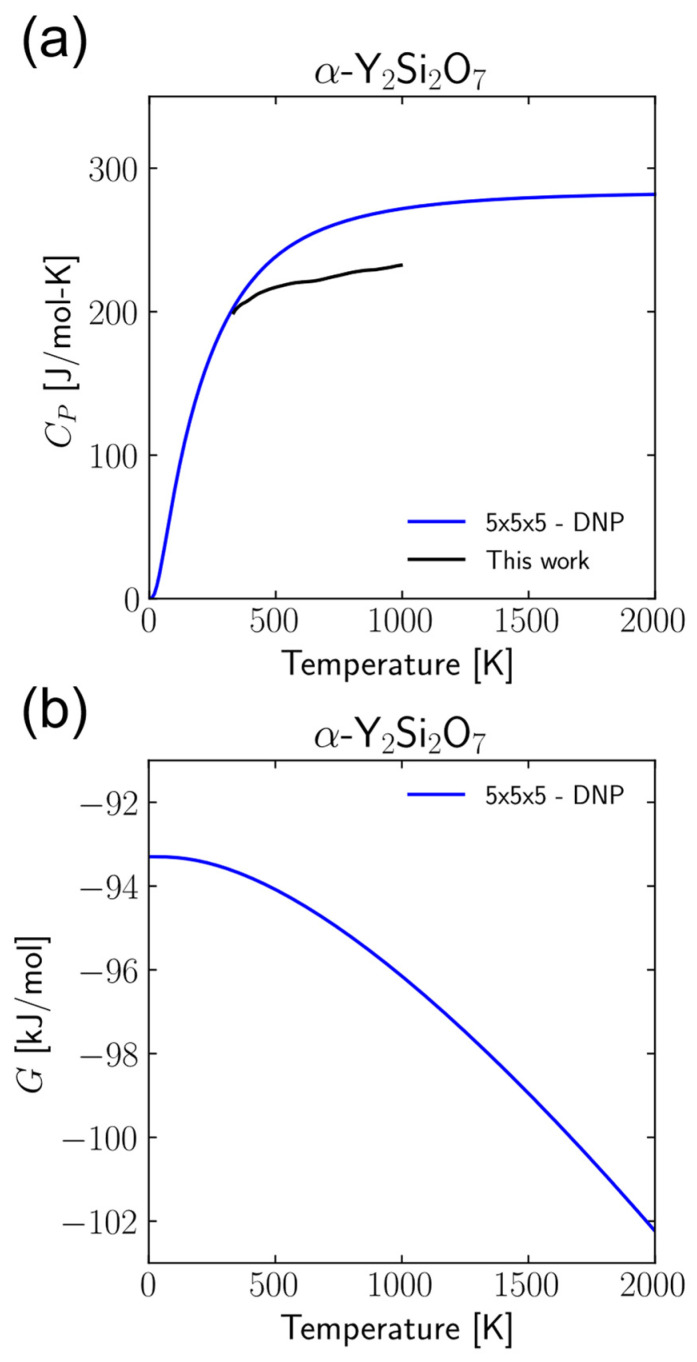
(**a**) Constant pressure heat capacities, *C_P_*, and (**b**) Gibbs free energy, *G*, for α-Y_2_Si_2_O_7_ calculated from DNP finite-displacement phonon calculations compared to experimental results.

**Figure 6:**
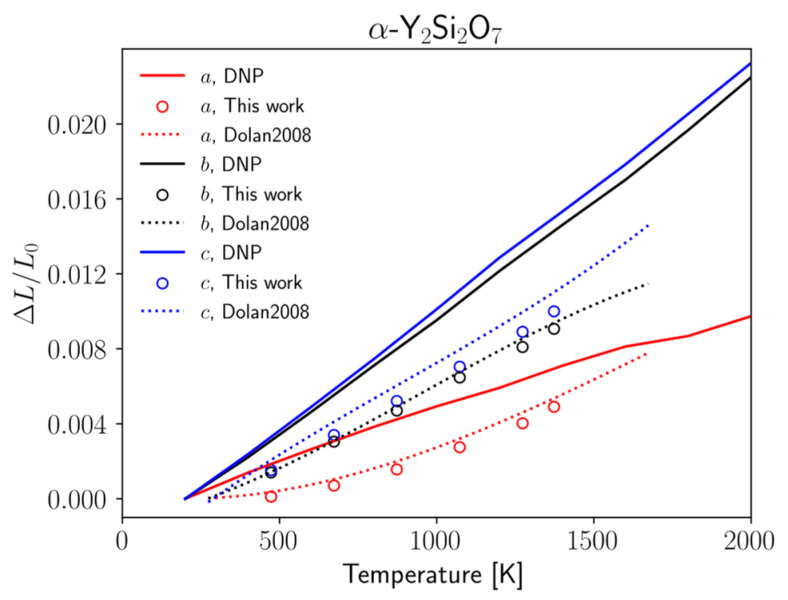
Thermal expansion of the α-Y_2_Si_2_O_7_ calculated from DNP trajectories. The dotted curve represents the fitted model from Dolan et al. [73]. Circle markers represent experimental data from this work.

Additionally, the thermodynamics properties calculated above were also calculated for α-Y_2_Si_2_O_7_. These results are shown in Figure 5 and Figure 6 and Table 4. The *C_p_* predicted by the DNP (Figure 5a) is very close to that predicted by the DNP for the other three crystal structures in this work, but there is significant deviation from that determined from the DSC measurements here. Further study is required to determine the accuracy of these calculations. There is a similar discrepancy in the calculated CTE (Figure 6) compared to the XRD measurements. In this case, the qualitative trends in the CTE anisotropy are correctly predicted, but the quantitative values are less accurate than the three crystal structures included in the DNP training dataset. These discrepancies likely indicate that a more thorough training process is required for a more transferrable DNP model. Further validation of the DNP on additional crystal structures with similar moieties to Y_2_Si_2_O_7_ such as yttrium monosilicate, yttria, and silica would provide additional evidence of model transferability and will be the subject of a future study.

## 4. Conclusions

In this work, we have developed an interatomic potential for Y_2_Si_2_O_7_ using a machine learning framework trained on DFT data. This potential was used in classical MD simulations to accurately reproduce thermodynamic properties calculated via finite-difference method phonon calculations. While certain properties, especially the calculated interatomic bond lengths, differed from the experimental results, the qualitative trends matched well, and the DNP was still able to predict properties of interest for the EBC design accurately. These results obtained orders of magnitude faster than state-of-the-art DFT calculations and could therefore be used as a screening method prior to more costly calculations or experiments. In addition, we used the potential to calculate the thermal expansion of three predominant crystal phases of Y_2_Si_2_O_7_. These simulated results showed the inherent anisotropy of the thermal expansion in each of the individual lattice directions. Finally, to test the transferability of the DNP, we calculated the properties for α-Y_2_Si_2_O_7_. The calculated results showed qualitative agreement with the trends from the experimental data, but a more robust training is likely needed for quantitative agreement for structures outside of the training dataset. The development of the DNP in this work enables future calculations of the material properties for Y_2_Si_2_O_7_ using alternative statistical sampling techniques such as Monte Carlo simulations or advanced MD sampling. These could be applied to additional properties of interest such as phase stability or gas diffusivity that would be computationally intractable with standard DFT methods. The results from these simulations could provide insights and guide the experimental validation of properties for which experimental data are scarce. Calculations of such properties using the DNP will be the subject of future studies. As such, the DNP developed in this work can be used to aid in and accelerate the computational design of EBCs for aerospace applications, thermal barrier coatings for aerospace and land-based power generation, coatings for nuclear thermal propulsion applications, and other related applications as well.

## Figures and Tables

**Table 1:** Calculated cell parameters for the β-, γ-, and δ-Y_2_Si_2_O_7_ conventional unit cell at 0 K. PBE is the Perdew–Burke–Ernzerhof exchange–correlation functional, PBEsol is the PBE revised for solids exchange–correlation functional. Experimental values were measured at 298 K [72,73,74,75,76,77,78,79].

Phase	Theory	a	b	c	α	β	γ
β C2/m	DFT (PBE)	6.914	9.063	4.781	90	101.97	90
	DFT (PBEsol)	6.854	8.965	4.740	90	101.79	90
	DNP (PBE *)	6.898	9.047	4.768	90	101.95	90
	Ref. [72]	6.88	8.97	4.72	90	101.7	90
	Ref. [78]	6.873	8.970	4.718	90	101.72	90
γ	DFT (PBE)	4.753	10.901	5.633	90	96.18	90
P2_1_/c	DFT (PBEsol)	4.710	10.813	5.569	90	95.98	90
	DNP (PBE *)	4.739	10.885	5.621	90	96.14	90
	Ref. [73]	4.68824 (5)	10.84072 (9)	5.58219 (6)	90	96.0325 (3)	90
	Ref. [74]	4.69	10.86	5.59	90	96.01	90
	Ref. [75]	4.663 (5)	10.784 (21)	5.536 (5)	90	96.06	90
	Ref. [76]	4.6881 (2)	10.8416 (5)	5.5824 (2)	90	96.035 (1)	90
	Ref. [78]	4.685	10.842	5.583	90	96.046	90
	Ref. [79]	4.6916 (4)	10.8521 (10)	5.5872 (5)	90	96.040 (3)	90
δ	DFT (PBE)	13.802	5.087	8.196	90	90	90
Pna2_1_	DFT (PBEsol)	13.619	5.027	8.121	90	90	90
	DNP (PBE *)	13.772	5.074	8.186	90	90	90
	Ref. [75]	13.69 (2)	5.020 (5)	8.165 (10)	90	90	90
	Ref. [77]	13.81	5.02	8.30	90	90	90
	Ref. [78]	13.663	5.020	8.150	90	90	90

* DNP was trained with AIMD simulations using the PBE functional.

**Table 2:** Coefficients of thermal expansion (×10^−6^ K^−1^) for β-, γ-, and δ-Y_2_Si_2_O_7_, including those for the individual lattice constants and for the average linear bulk CTE.

Phase		T Range [K]	*a* (×10^−6^ K^−1^)	*b* (×10^−6^ K^−1^)	*c* (×10^−6^ K^−1^)	Average Bulk Linear CTE (×10^−6^ K^−1^)
β C2/m	DNP	200–2000	7.45	4.98	0.89	4.33
	Ref. [24]	298.15–1773.15	8.13	4.66	1.41	4.73
	Ref. [73]	293.15–1473.15 293.15–1673.15	5.74 6.23	4.17 4.37	2.23 2.12	4.0 4.1
γ P2_1_/c	DNP	200–2000	6.89	6.83	0.79	4.77
	Ref. [24]	298.15–1773.15	6.14	6.67	1.11	4.64
	Ref. [73]	293.15–1473.15	0.69	5.90	5.17	3.9
	Ref. [76]	504				2.2
	Ref. [76]	1473				3.8
	Ref. [86]	293–1527				3.9
	Ref. [90]	293–1273				4.6
δ Pna2_1_	DNP	200–2000	11.80	11.48	3.92	9.20
	Ref. [24]	298.15–1773.15	11.22	10.43	2.41	8.02
	Ref. [73]	293.15–1473.15 293.15–1673.15	10.40 10.75	9.92 10.32	2.48 2.59	7.7 8.1

**Table 3:** Calculated cell parameters for the α-Y_2_Si_2_O_7_ conventional unit cell at 0 K. PBE is the Perdew–Burke–Ernzerhof exchange–correlation functional, PBEsol is the PBE revised for solids exchange–correlation functional. Experimental values were measured at 298 K [72,73,74,75,76,77,78,79].

Phase	Theory	a	b	c	α	β	Γ
α $P\bar{1}$	DFT (PBE)	6.660	6.694	12.153	94.07	91.53	92.11
	DFT (PBEsol)	6.593	6.616	12.00	94.47	91.08	91.84
	DNP (PBE *)	6.655	6.693	12.162	94.50	91.21	91.89
	Expt. ^†^	6.581	6.633	12.021	94.53	89.03	88.23
	Ref. [73]	6.5881(4)	6.6392(6)	12.031(1)	94.479(7)	90.946(7)	91.800(6)

* DNP was trained with AIMD simulations using the PBE functional. ^†^ This work.

**Table 4:** Coefficients of thermal expansion (×10^−6^ K^−1^) for α-Y_2_Si_2_O_7_, including those for the individual lattice constants and for the average linear bulk CTE.

Phase		T Range [K]	*a* (×10^−6^ K^−1^)	*b* (×10^−6^ K^−1^)	*c* (×10^−6^ K^−1^)	Average Bulk Linear CTE (×10^−6^ K^−1^)
α $P\bar{1}$	DNP	200–2000	5.36	12.47	12.95	10.77
	Expt.^†^	298.15–1373.15	5.36	8.49	9.33	7.72
	Ref. [73]	293.15–1473.15	5.21	8.60	10.29	8.0

^†^ This work.

## Data Availability

The data presented in this study are available on request from the corresponding author.

## Supplementary Materials

The following supporting information can be downloaded at: https://www.mdpi.com/article/10.3390/ma17020286/s1, Figure S1: β-Y_2_Si_2_O_7_ radial distribution function from 0 to 10 Å; Figure S2: γ-Y_2_Si_2_O_7_ radial distribution function from 0 to 10 Å; Figure S3: δ-Y_2_Si_2_O_7_ radial distribution function from 0 to 10 Å; Figure S4: Heat capacities (*C_P_*) calculated via the QHA for β-Y_2_Si_2_O_7_ using DFT (including the PBE and PBEsol exchange-correlation functionals) and classical MD and the DNP as the respective force calculators. For the DNP simulations, we compared supercells of size *N*×*N*×*N* where *N* = 1, 2, 3, and 5; Figure S5: Gibbs free energies (*G*) calculated via the QHA for β-Y_2_Si_2_O_7_ using DFT (including the PBE and PBEsol exchange-correlation functionals) and classical MD and the DNP as the respective force calculators. For the DNP simulations, we compared supercells of size *N*×*N*×*N* where *N* = 1, 2, 3, and 5; Figure S6: Heat capacities (*C_P_*) calculated via the QHA for γ-Y_2_Si_2_O_7_ using DFT (including the PBE and PBEsol exchange-correlation functionals) and classical MD and the DNP as the respective force calculators. For the DNP simulations, we compared supercells of size *N*×*N*×*N* where *N* = 1, 2, 3, and 5; Figure S7: Gibbs free energies (*G*) calculated via the QHA for γ-Y_2_Si_2_O_7_ using DFT (including the PBE and PBEsol exchange-correlation functionals) and classical MD and the DNP as the respective force calculators. For the DNP simulations, we compared supercells of size *N*×*N*×*N* where *N* = 1, 2, 3, and 5; Figure S8: Heat capacities (*C_P_*) calculated via the QHA for δ-Y_2_Si_2_O_7_ using DFT (including the PBE and PBEsol exchange-correlation functionals) and classical MD and the DNP as the respective force calculators. For the DNP simulations, we compared supercells of size *N*×*N*×*N* where *N* = 1, 2, 3, and 5; Figure S9: Gibbs free energies (*G*) calculated via the QHA for δ-Y_2_Si_2_O_7_ using DFT (including the PBE and PBEsol exchange-correlation functionals) and classical MD and the DNP as the respective force calculators. For the DNP simulations, we compared supercells of size *N*×*N*×*N* where *N* = 1, 2, 3, and 5; Figure S10: Anisotropic coefficients of thermal expansion for the *a* (red), *b* (black), and *c* (blue) directions of β-Y_2_Si_2_O_7_. Increasing *N* is represented by increasing dash opacity and length. Ref. [73] indicates the empirical fit by Dolan, et al. [2]; Figure S11: Anisotropic coefficients of thermal expansion for the *a* (red), *b* (black), and *c* (blue) directions of γ-Y_2_Si_2_O_7_. Increasing *N* is represented by increasing dash opacity and length. Ref. [73] indicates the empirical fit by Dolan, et al. [2]; Figure S12: Anisotropic coefficients of thermal expansion for the *a* (red), *b* (black), and *c* (blue) directions of δ-Y_2_Si_2_O_7_. Increasing *N* is represented by increasing dash opacity and length. Ref. [73] indicates the empirical fit by Dolan, et al. [2].
