# Supplementary material for: Theoretical Prediction of Thermal Expansion Anisotropy for Y2Si2O7 Environmental Barrier Coatings Using a Deep Neural Network Potential and Comparison to Experiment
Source: Materials (Basel). 2024 Jan 5;17(2):286. doi: 10.3390/ma17020286 (PMC10817232; doi:10.3390/ma17020286)
Supplement: Supplementary file 1 [file materials-17-00286-s001.zip › materials-2746627-supplementary.pdf]

Supplemental Information

1 Radial distribution functions

Full RDF plots for the 5x5x5 supercells of β-, γ-, and δ-$Y_2Si_2O_7$ from 200 K to 2000 K in increments of 200 K. These plots show that the height and sharpness of the bond peaks decrease and broaden with increasing temperature, indicative of greater fluctuation in bond distances. However, for all three crystal structures, there are still bond peaks present even out to a cutoff distance of 10 Å. Therefore, we determine that the crystal structures do not indicate melting over the course of the molecular dynamics trajectories. This agrees with the experimentally determined melting temperature of $Y_2Si_2O_7$ of ~2100 K.[1]

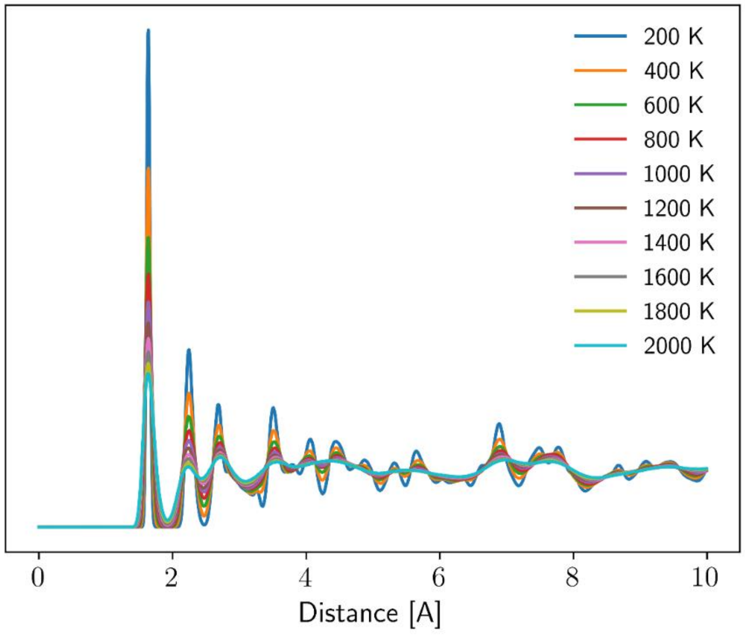

Figure S1: β-$Y_2Si_2O_7$ radial distribution function from 0 to 10 Å.

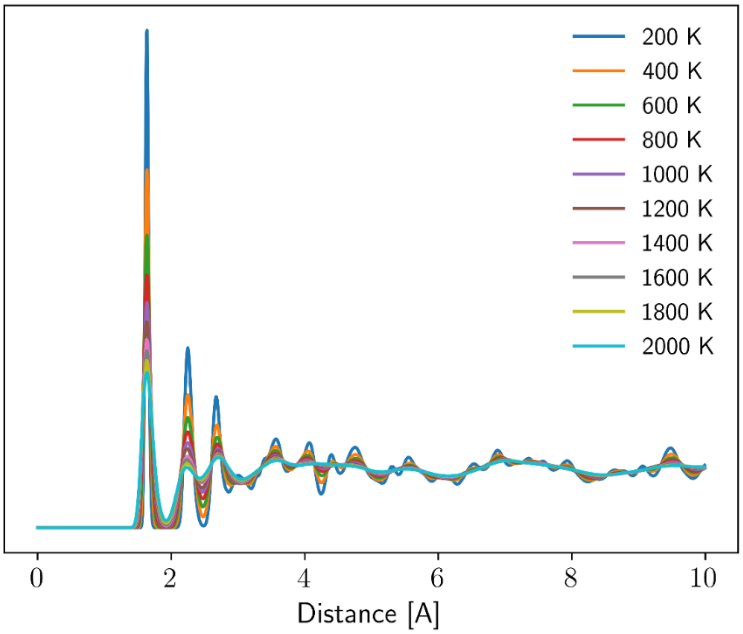

Figure S2: γ-$Y_2Si_2O_7$ radial distribution function from 0 to 10 Å.

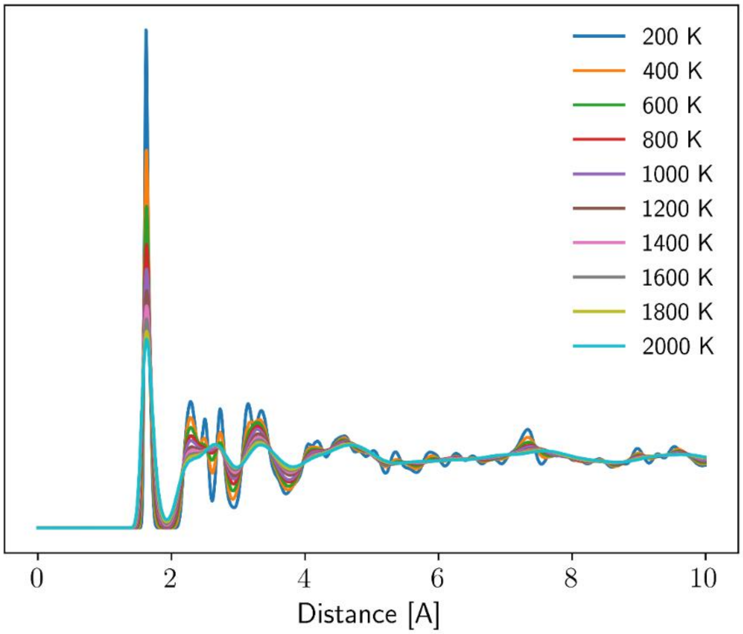

Figure S3: δ-$Y_2Si_2O_7$ radial distribution function from 0 to 10 Å.

2 Unit cell size effects

2.1 Heat capacities and Gibbs free energies

The calculated heat capacities and Gibbs free energies for β-, γ-, and δ-$Y_2Si_2O_7$ as a function of temperature and calculation method are shown in Figures S4-S9. DFT calculations were performed using both the PBE and PBEsol exchange-correlation functionals. As stated in the main text, the Deep Neural Network Potential (DNP) was trained using DFT ab initio simulations using the PBE functional. For the quasi-harmonic approximation (QHA) phonon calculations, all DFT calculations were performed with 2x2x2 supercells of the respective conventional crystal structures as indicated in the main text. QHA calculations utilizing classical molecular dynamics force calculations via the DNP were performed using a range of $N \times N \times N$ supercells where $N = 1, 2, 3$, and 5.

For all crystal structures considered, there was significant deviation in the Gibbs free energy between the DFT calculated with the PBE functional compared to those with the PBEsol functional. There were also deviations in the calculated heat capacities between the two functionals. Interestingly, there was generally better agreement between the PBE heat capacities and experimental results than the PBEsol heat capacities, as shown in Figure 3 in the main text.

For the different supercell sizes, we generally saw that the calculated Gibbs free energies and heat capacities predicted by the QHA and DNP tended to converge at $N = 2$. There were still discrepancies between the DFT and DNP results even at $N = 2$. However, as shown in Figure 3 in the main text, the DNP tended to predict heat capacities closer to experimental values even compared to DFT.

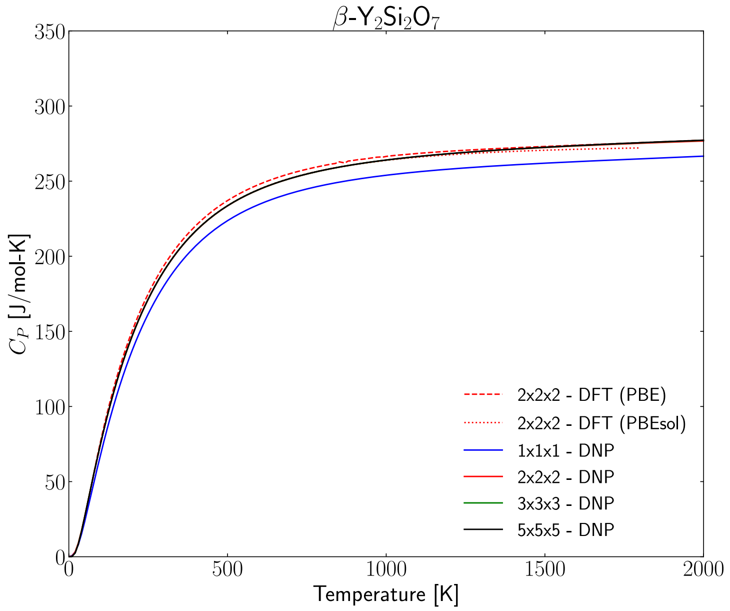

Figure S4: Heat capacities (C_p) calculated via the QHA for β-$Y_2Si_2O_7$ using DFT (including the PBE and PBEsol exchange-correlation functionals) and classical MD and the DNP as the respective force calculators. For the DNP simulations, we compared supercells of size $N \times N \times N$ where N = 1, 2, 3, and 5.

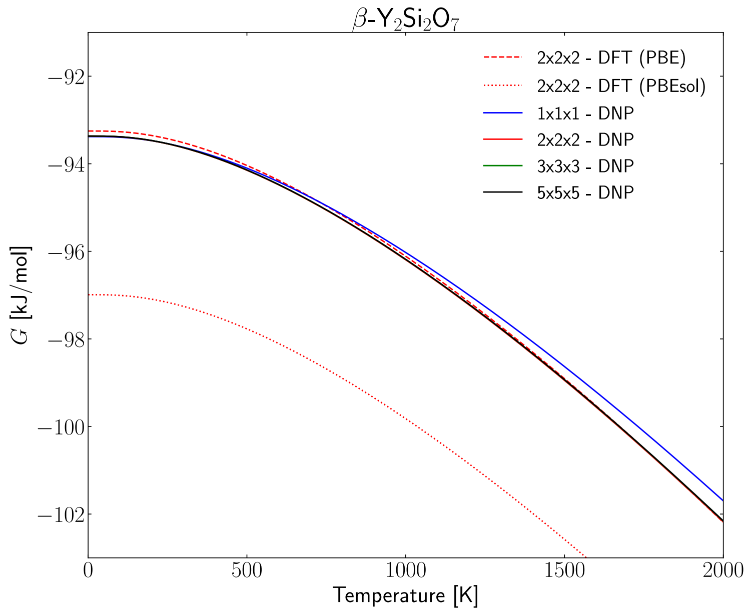

Figure S5: Gibbs free energies (G) calculated via the QHA for β-$Y_2Si_2O_7$ using DFT (including the PBE and PBEsol exchange-correlation functionals) and classical MD and the DNP as the respective force calculators. For the DNP simulations, we compared supercells of size $N \times N \times N$ where N = 1, 2, 3, and 5.

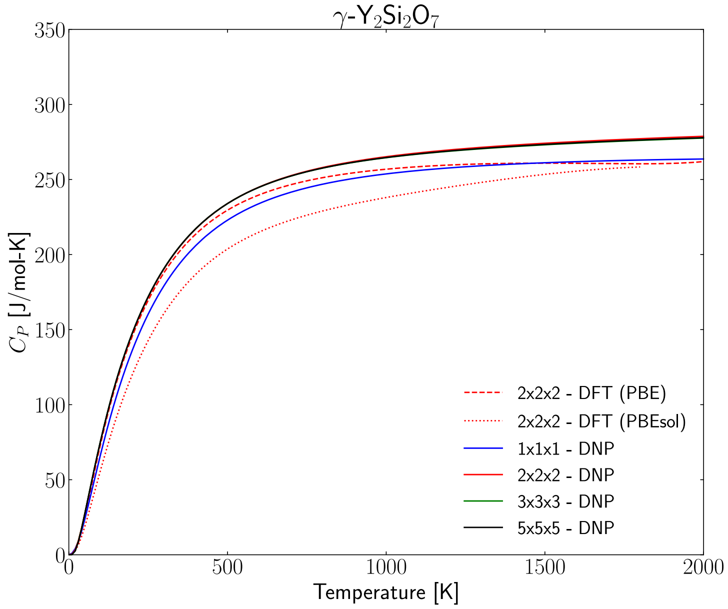

Figure S6: Heat capacities (C_p) calculated via the QHA for γ-$Y_2Si_2O_7$ using DFT (including the PBE and PBEsol exchange-correlation functionals) and classical MD and the DNP as the respective force calculators. For the DNP simulations, we compared supercells of size *N*×*N*×*N* where *N* = 1, 2, 3, and 5.

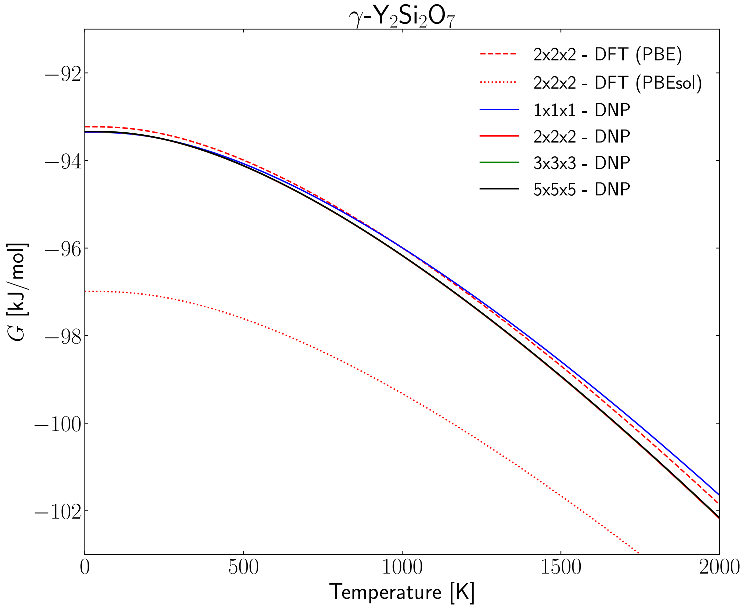

Figure S7: Gibbs free energies (*G*) calculated via the QHA for γ-$Y_2Si_2O_7$ using DFT (including the PBE and PBEsol exchange-correlation functionals) and classical MD and the DNP as the respective force calculators. For the DNP simulations, we compared supercells of size *N*×*N*×*N* where *N* = 1, 2, 3, and 5.

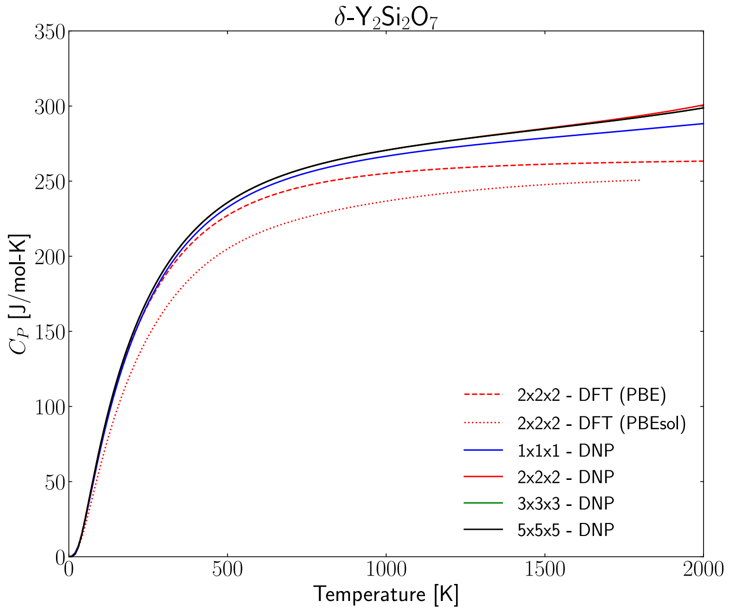

Figure S8: Heat capacities (C_p) calculated via the QHA for δ-$Y_2Si_2O_7$ using DFT (including the PBE and PBEsol exchange-correlation functionals) and classical MD and the DNP as the respective force calculators. For the DNP simulations, we compared supercells of size *N*×*N*×*N* where *N* = 1, 2, 3, and 5.

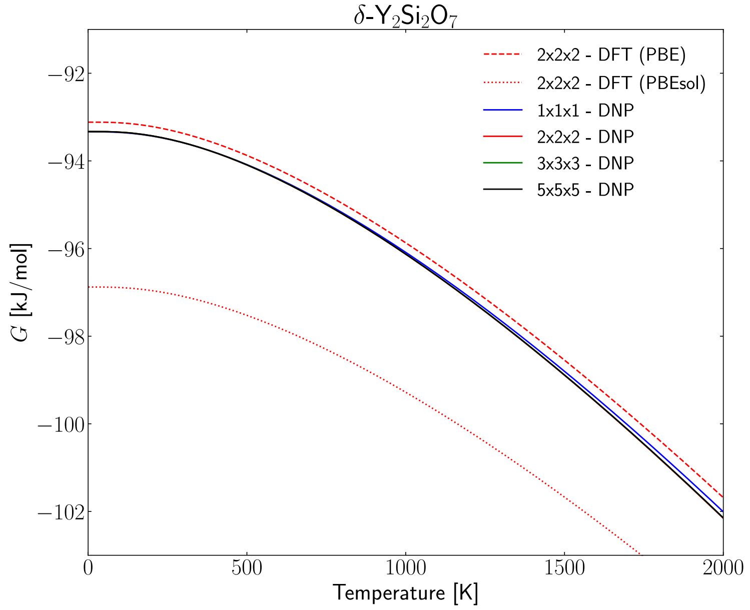

Figure S9: Gibbs free energies (G) calculated via the QHA for δ-$Y_2Si_2O_7$ using DFT (including the PBE and PBEsol exchange-correlation functionals) and classical MD and the DNP as the respective force calculators. For the DNP simulations, we compared supercells of size *N*×*N*×*N* where *N* = 1, 2, 3, and 5.

2.2 Coefficients of thermal expansion

Anisotropic coefficients of thermal expansion were calculated as a function of the supercell size for *N* = 1, 2, 3, and 5. These results are shown in Figures S10-S12. The empirical fits from Dolan, et al. [2] (Reference 68 in the main text) are used here to approximate the true values since they generally match the other literature data well and can be extrapolated to fit the modeled temperature range. The temperatures have been referenced to the initial temperature, which was 200 K for the simulated results in this work and 293.15 K for the empirical fits by Dolan, et al., for the sake of a more direct comparison due to the differing initial temperatures. The results indicate that the calculated CTE values generally more closely approximate the experimental data as *N* increases. Additionally, the calculation results do appear to be converging with respect to *N*. While it is possible, or even likely, that the calculated results would improve with even further increasing *N*, a full analysis of the impacts of unit cell size was beyond the scope of this study.

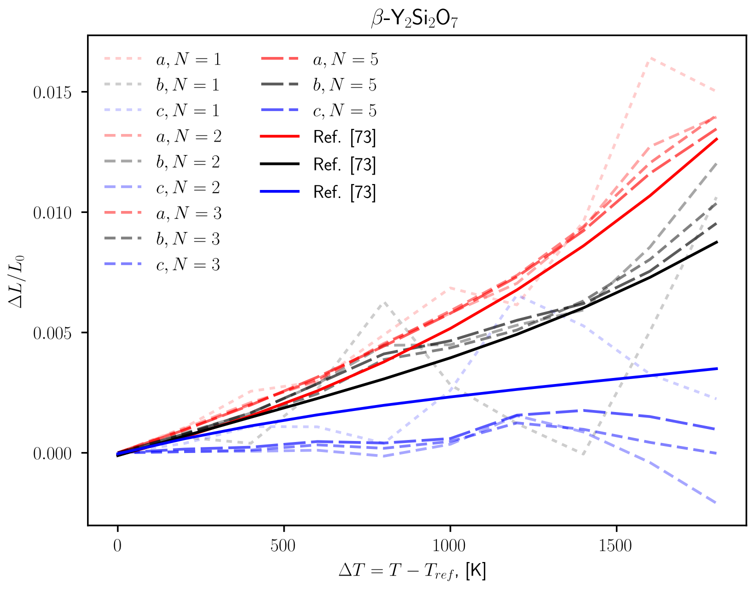

Figure S10: Anisotropic coefficients of thermal expansion for the a (red), b (black), and c (blue) directions of β-$Y_2Si_2O_7$. Increasing *N* is represented by increasing dash opacity and length. Ref. [73] indicates the empirical fit by Dolan, et al. [2] (Reference 73 in the main text).

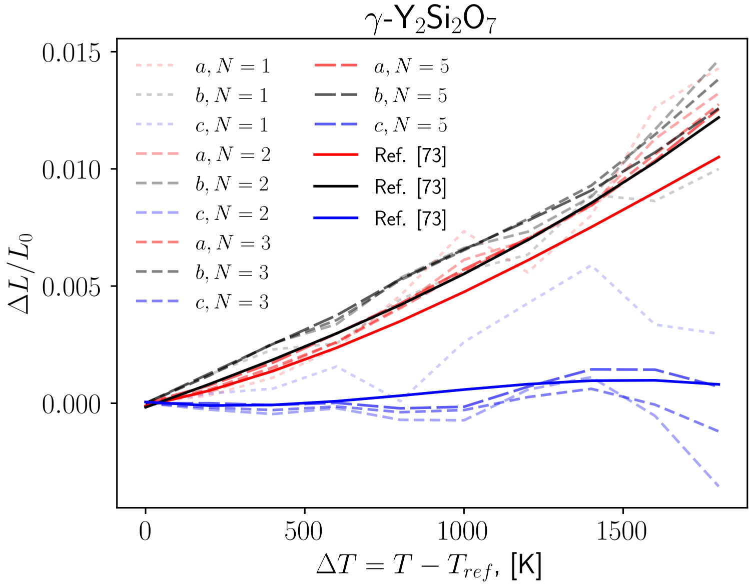

Figure S11: Anisotropic coefficients of thermal expansion for the a (red), b (black), and c (blue) directions of γ-Y2Si2O7. Increasing *N* is represented by increasing dash opacity and length. Ref. [73] indicates the empirical fit by Dolan, et al. [2] (Reference 73 in the main text).

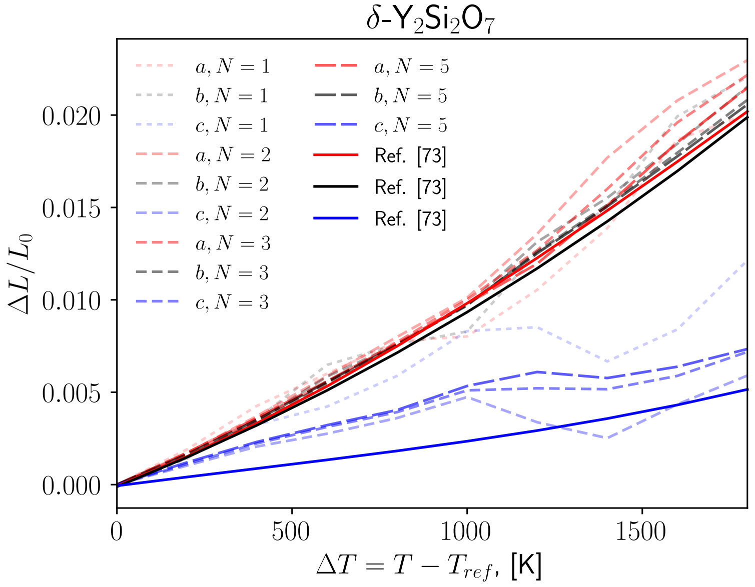

Figure S12: Anisotropic coefficients of thermal expansion for the a (red), b (black), and c (blue) directions of δ-Y2Si2O7. Increasing *N* is represented by increasing dash opacity and length. Ref. [73] indicates the empirical fit by Dolan, et al. [2] (Reference 73 in the main text).

3 References

[1] Mao, H.; Selleby, M.; Fabrichnaya, O. Thermodynamic Reassessment of the Y2O3-Al2O3-SiO2 System and Its Subsystems. *Calphad Comput. Coupling Phase Diagrams Thermochem.* **2008**, *32* (2), 399–412. https://doi.org/10.1016/j.calphad.2008.03.003.

[2] Dolan, M. D.; Harlan, B.; White, J. S.; Hall, M.; Misture, S. T.; Bancheri, S. C.; Bewlay, B. Structures and Anisotropic Thermal Expansion of the α, β, γ, and δ Polymorphs of Y2Si2O7. *Powder Diffr.* **2008**, *23* (1), 20–25. https://doi.org/10.1154/1.2825308.
